# Incidental Large Deep Femoral Artery Aneurysm Successfully Treated With Autologous Vein Bypass Reconstruction: A Case Report

**DOI:** 10.7759/cureus.107861

**Published:** 2026-04-28

**Authors:** Shuhei Kawamoto, Shunya Ono, Tabata Kokoro, Kosaku Nishigawa, Takeyuki Kanemura

**Affiliations:** 1 Cardiothoracic Surgery, IMS Katsushika Heart Center, Tokyo, JPN; 2 Cardiovascular Surgery, IMS Katsushika Heart Center, Tokyo, JPN

**Keywords:** collateral circulation, deep femoral artery aneurysm, revascularization, saphenous vein graft, vascular surgery

## Abstract

Deep femoral artery (DFA) aneurysms are rare vascular lesions that may remain asymptomatic until they enlarge or rupture. We report a case of a 74-year-old man with an incidentally detected large DFA aneurysm measuring 41 mm, discovered during evaluation for left lower limb deep venous thrombosis (DVT). The patient underwent revascularization with a common femoral artery (CFA)-to-deep femoral artery bypass using an autologous great saphenous vein graft. Postoperative contrast-enhanced computed tomography (CECT) confirmed graft patency, and the patient recovered without complications. Histopathology revealed atherosclerotic changes with cystic medial degeneration. This case highlights the clinical significance of early detection and the advantages of autologous vein graft reconstruction.

## Introduction

True aneurysms of the deep femoral artery (DFA) are extremely rare, representing approximately 0.5% of all peripheral arterial aneurysms and 1%-2.6% of femoral artery aneurysms [1,2]. Because the DFA lies deep within the thigh musculature, has a thick muscular wall, and plays an important role in collateral perfusion of the lower limb, aneurysm formation is uncommon [3]. However, once an aneurysmal change occurs, the risk of rupture is significant and may result in life-threatening hemorrhage [4].

Most DFA aneurysms are asymptomatic and are often detected incidentally [2,5]. The optimal management strategy remains controversial due to the limited number of reported cases and includes options such as ligation, endovascular treatment, and surgical reconstruction [6]. We report a case of a large DFA aneurysm successfully treated with autologous vein bypass reconstruction.

## Case presentation

**Figure 1:**
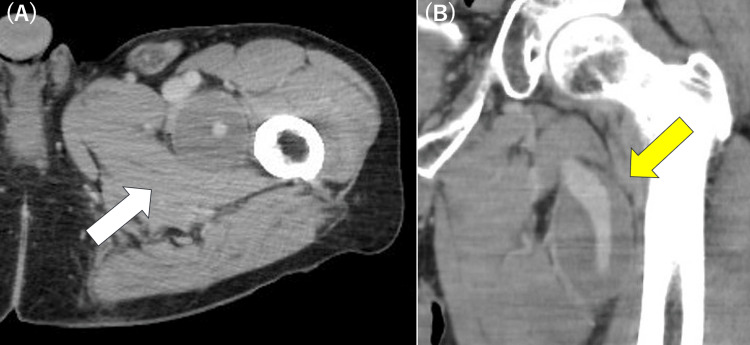
Preoperative CECT images (A) Axial view showing a deep femoral artery aneurysm (white arrow). (B) Coronal view demonstrating the aneurysm (yellow arrow), with a maximum longitudinal diameter of 69 mm. CECT: contrast-enhanced computed tomography

A 74-year-old man was referred to our department after an incidental finding of a left deep femoral artery aneurysm during evaluation for peripheral deep venous thrombosis (DVT) at another hospital. The patient denied any symptoms, including thigh pain, swelling, a palpable pulsatile mass, or signs of limb ischemia. The deep venous thrombosis (DVT) was located in the same left lower limb and was classified as peripheral DVT. The patient was managed conservatively with direct oral anticoagulant (DOAC) therapy. The patient had a history of hypertension and a significant smoking history (20 cigarettes per day from age 20 to 53, with smoking cessation 11 years prior to surgery), both of which are well-known risk factors for atherosclerotic disease. Contrast-enhanced computed tomography (CECT) demonstrated a 41-mm aneurysm of the left DFA with a longitudinal diameter of 69 mm (Figure 1).

On admission, his vital signs were stable, except for an irregular pulse of 110 beats per minute, which was attributed to paroxysmal atrial fibrillation based on clinical evaluation. Peripheral pulses were detectable by Doppler ultrasonography. The preoperative ankle-brachial index was 1.13 on the right and 0.94 on the left. Laboratory findings showed no significant abnormalities, and echocardiography revealed preserved left ventricular function.

**Figure 2:**
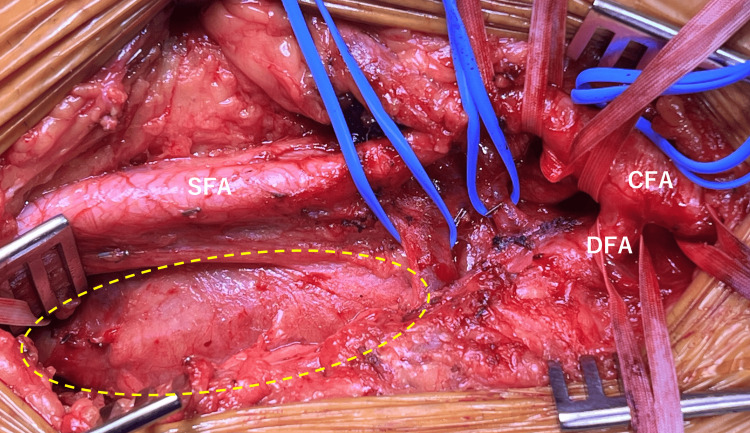
Intraoperative exposure of the left deep femoral artery aneurysm A deep femoral artery aneurysm (yellow dotted line) located after the bifurcation. The top of the image represents the operator’s side, the right side represents the patient’s cranial side, and the left side represents the patient’s caudal side. CFA: common femoral artery, SFA: superficial femoral artery, DFA: deep femoral artery

Surgical intervention was planned due to the size of the aneurysm. Through a longitudinal incision along the medial aspect of the thigh, the common femoral artery (CFA), superficial femoral artery (SFA), and DFA were exposed. The aneurysm was identified distal to the bifurcation (Figure 2).

**Figure 3:**
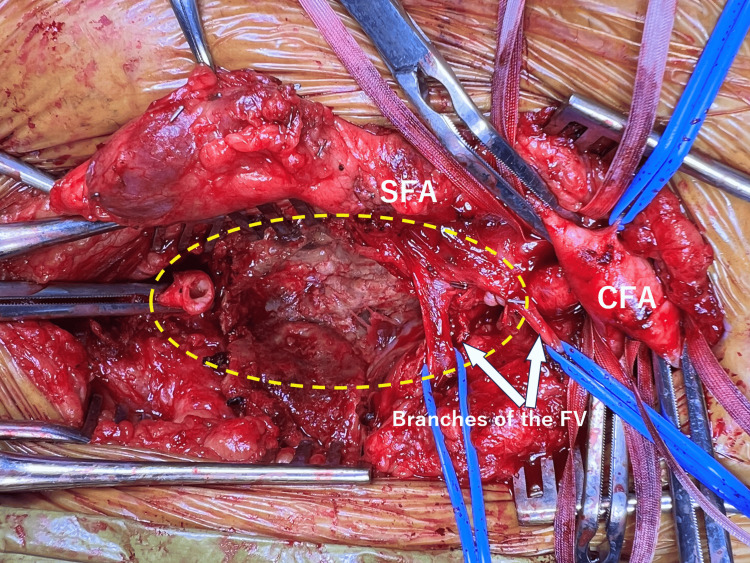
Intraoperative view of aneurysm sac opening The aneurysm sac is opened, and organized thrombus is removed (yellow dotted line). CFA: common femoral artery, SFA: superficial femoral artery, FV: femoral vein

After systemic heparinization with an initial dose of 5,000 units, proximal and distal control was achieved. Activated clotting time (ACT) was monitored and maintained at approximately 300 seconds during the procedure. The aneurysm sac was opened, revealing organized thrombus, which was removed (Figure 3).

**Figure 4:**
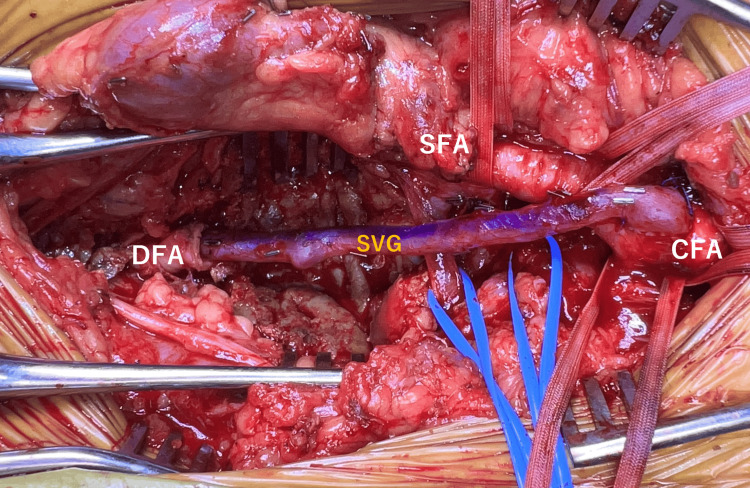
Intraoperative view of bypass reconstruction Bypass reconstruction using an autologous great saphenous vein graft between the common femoral artery and the deep femoral artery. CFA: common femoral artery, SFA: superficial femoral artery, DFA: deep femoral artery, SVG: saphenous vein graft

Multiple branch vessels arising from the aneurysm were identified and ligated. A segment of the great saphenous vein was harvested and used for bypass reconstruction. The proximal anastomosis was performed in an end-to-side fashion to the CFA, and the distal anastomosis was performed end-to-end to the DFA (Figure 4). Both anastomoses were performed using 6-0 Surgipro II polypropylene sutures. The total operative time was 5 hours and 48 minutes. The arterial clamp time was not recorded; however, no intraoperative complications were observed. The great saphenous vein graft was used in a reversed configuration. The graft measured approximately 12 cm in length and 3 mm in diameter. The diameter of the common femoral artery at the proximal anastomotic site was 10.7 mm, and the diameter of the deep femoral artery at the distal anastomotic site was 6.7 mm. Although there was a size discrepancy between the graft and native vessels, no technical difficulty was encountered, and satisfactory graft flow was confirmed intraoperatively.

**Figure 5:**
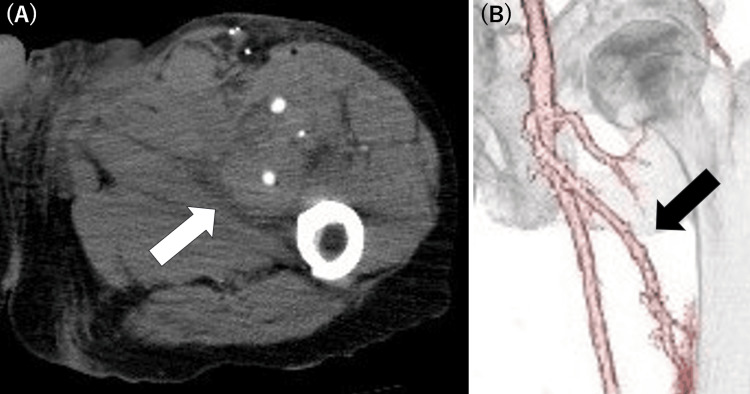
Postoperative CECT images (A) Axial view showing the patent graft (white arrow). (B) Three-dimensional reconstruction demonstrating successful bypass with good graft patency (black arrow). CECT: contrast-enhanced computed tomography

Postoperatively, the patient had an uneventful recovery. The ankle-brachial index improved to 1.23 on the right and 1.14 on the left, and follow-up CECT demonstrated good graft patency (Figure 5).

The patient was discharged on postoperative day 19. Histopathological examination of the aneurysm wall revealed atherosclerosis with focal cystic medial degeneration, consistent with a degenerative true aneurysm.

## Discussion

Deep femoral artery (DFA) aneurysms are uncommon vascular entities, largely due to the artery’s anatomical protection within the deep muscular compartment of the thigh and its relatively thick medial layer [3,7]. These structural features are believed to reduce exposure to hemodynamic stress and atherosclerotic degeneration compared to more superficial arteries. Nevertheless, once aneurysmal change develops, DFA aneurysms are associated with a considerable risk of rupture, thrombosis, and distal embolization, which may result in acute limb ischemia or life-threatening hemorrhage [4,8].

Isolated true deep femoral artery aneurysms are exceptionally rare, accounting for approximately 0.5% of all peripheral arterial aneurysms and 1%-2.6% of all femoral artery aneurysms [1,2]. Due to their deep anatomical location, these aneurysms often remain asymptomatic until they reach a large size, with a relatively high risk of rupture reported to be between 18% and 45% [4,8]. The insidious nature of this condition often leads to delayed diagnosis, and many cases are detected incidentally during imaging performed for other conditions.

Computed tomography angiography (CTA) remains the gold standard for the definitive diagnosis of deep femoral artery aneurysms. It allows precise evaluation of vessel size and provides important information regarding intraluminal thrombus. In contrast, ultrasound and magnetic resonance imaging may have limitations in distinguishing partially thrombosed aneurysms from soft tissue tumors [9].

In the present case, the deep femoral artery aneurysm was detected during evaluation for DVT in the same limb. The DVT was classified as peripheral and was managed conservatively with anticoagulation therapy. Although a direct causal relationship cannot be definitively established, the aneurysm may have contributed to venous compression and impaired venous return, potentially facilitating thrombus formation [10].

An important clinical consideration is the high prevalence of concomitant aneurysmal disease in patients with DFA aneurysms. Previous studies have reported that 65%-75% of patients harbor additional aneurysms in other vascular territories, particularly in the abdominal aorta and popliteal arteries [4,11]. This highlights the necessity of comprehensive vascular imaging once a DFA aneurysm is identified. Although no additional aneurysms were detected in our patient, the presence of multiple arterial pathologies should always be considered, especially in elderly patients with atherosclerotic risk factors.

In addition to systemic atherosclerotic risk factors, smoking may contribute to aneurysmal degeneration through molecular mechanisms. Experimental studies in abdominal aortic aneurysm models have shown that nicotine can activate AMP-activated protein kinase α2 in vascular smooth muscle cells, leading to increased expression of matrix metalloproteinase-2, which promotes degradation of the vascular wall [12].

The indications for surgical intervention remain controversial due to the rarity of the disease and the lack of large-scale studies. While some authors suggest a diameter threshold of 25 mm for intervention, others advocate early repair regardless of size, citing the unpredictable natural history and the potential for catastrophic rupture [5,13]. In the present case, the aneurysm measured 41 mm, clearly exceeding commonly accepted thresholds, thereby justifying surgical treatment.

Surgical treatment strategies for deep femoral artery aneurysms include aneurysm resection with reconstruction using a prosthetic or autologous vein graft, simple ligation, and endovascular embolization, which is particularly applicable to distally located lesions where reconstruction may be less feasible. There is currently no consensus on the optimal approach [6]. However, the deep femoral artery serves as an important collateral pathway, especially in patients with superficial femoral artery occlusion. Therefore, reconstruction with preservation of blood flow is often desirable when anatomically feasible. In contrast, simple ligation or distal embolization may be considered for aneurysms located more distally or when reconstruction is not technically suitable. The choice of treatment strategy should be individualized based on anatomical and clinical factors. Although ligation alone has been reported to yield acceptable outcomes in selected cases, revascularization should be strongly considered to preserve limb perfusion and prevent ischemic complications.

In the present case, we elected to perform open surgical reconstruction using an autologous great saphenous vein graft. The choice of conduit remains a subject of debate, as both prosthetic grafts and autologous veins have demonstrated comparable patency rates [14]. However, autologous vein grafts offer distinct advantages, particularly in reducing the risk of graft infection, which is a critical concern in groin surgery [14,15].

Although autologous vein grafts are generally considered durable, aneurysmal degeneration of venous conduits has been reported, albeit rarely, particularly in the context of popliteal artery aneurysm repair. The underlying mechanisms remain unclear but may include progression of atherosclerosis, systemic dilating diathesis, graft varicosities, low-grade infection, and post-stenotic dilatation [16].

Postoperative complications such as lymphatic leakage, wound infection, and graft infection have been reported with variable frequency in femoral artery surgery [15]. Notably, our patient experienced an uncomplicated postoperative course.

## Conclusions

We report a rare case of a large deep femoral artery aneurysm successfully treated with autologous great saphenous vein bypass reconstruction. Given the potential role of the DFA as an important collateral pathway and the lower infection risk associated with autologous grafts, this approach may represent a safe and effective treatment option when anatomically feasible. When autologous vein grafting is planned, preoperative screening for deep vein thrombosis is also important to reduce the risk of postoperative venous thromboembolism and to ensure the suitability of the vein conduit. Further studies are needed to better define the optimal timing of intervention for asymptomatic deep femoral artery aneurysms. In addition, long-term follow-up is essential to evaluate graft durability and patency.
